# Advancements in the Application of Numerical Simulation During Tablet Compaction

**DOI:** 10.3390/pharmaceutics17020220

**Published:** 2025-02-08

**Authors:** Zhe Li, Haolong Xiong, Qiong Li, Abid Naeem, Lingyu Yang, Weifeng Zhu, Yanni Wu, Zhengji Jin, Liangshan Ming

**Affiliations:** 1 Key Laboratory of Modern Preparation of TCM of Ministry of Education, Institute for Advanced Study, Jiangxi University of Chinese Medicine, Nanchang 330004, China; lizhezd@163.com (Z.L.); xionghaolong1@jxutcm.edu.cn (H.X.); zwf0322@126.com (W.Z.); 2 School of Life Science, Advanced Research Institute of Multidisciplinary Science, College of Medical Technology, Key Laboratory of Molecular Medicine and Biotherapy, Key Laboratory of Medical Molecule Science and Pharmaceutics Engineering, Beijing Institute of Technology, Beijing, 100081, China; anaeemktk@gmail.com; 3 Jiangzhong Pharmaceutical Co., Ltd., Nanchang 330049, China; yly@crjz.com (L.Y.); 15870016839@163.com (Y.W.)

**Keywords:** numerical simulation technology, compaction, tablet, DEM, FEM

## Abstract

**Background:** Numerical simulation is a technique that utilizes electronic computers to combine concepts of the discrete element method (DEM), finite element method (FEM), computational fluid dynamics (CFD), etc., and express simulated behaviors utilizing numerical computations and images. Compaction is the main process of tablet manufacturing; most of the current studies have focused on macroscopic compaction and tablet characterization, while the internal stress state and microstructure changes as a result of the compaction process are not well understood. Therefore, an in-depth understanding of the flow and compaction behavior of pharmaceutical powders is essential for the analysis and control of the compaction process. **Methods:** Current research shows that compaction is shifting from macroscopic behavior toward internal microscopic behavior using numerical simulation technology. **Results:** This review focuses on the application of various numerical simulation technologies during compaction and the contact model, or the constitutive equation commonly used in numerical simulation. In addition, the difficulties of numerical simulation technology in calibrating powder parameters and the limitations of the current research are also discussed. **Conclusions:** Numerical simulation research in medicine and other fields will continue to flourish as numerical simulation technology advances, attracting more and more researchers using it effectively.

## 1. Introduction

Tablets are a common product in the food, health, chemical, and pharmaceutical industries [1]. The preparation process is divided into three phases: die filling, compaction, and ejection [2,3]. Firstly, the powder is filled into the die cavity using gravity or suction under the motion of the feed frame [4]. Secondly, the upper and lower punches in the die cavity move in opposite directions, which applies pressure to the powder, reduces the porosity, and compacts the powder with certain mechanical properties [5]. After compaction, the upper punch returns to its original position, and the lower punch ejects the compacted powder out of the die cavity [6]. Due to the differences in the properties of pharmaceutical powders, it is crucial to have an in-depth understanding of the compactibility of powders.

In the past, the compaction properties of powders have been mainly described as a function of relative density and external load [7], which is usually indicated by the Heckel equation, the Kawakita equation, the Walker equation, the Shapiro equation, and Cooper-Eaton’s equation, etc. The compaction process is where the pharmaceutical powder is subjected to an external load, which produces a rearrangement within the die cavity, resulting in a more dense stacking structure [8]. Subsequently, the particles are subjected to elastic and plastic deformation; the elastic deformation is reversible, and the plastic deformation is irreversible, which leads to particle breakage [9]. When the pressure on the particles exceeds the limit, the particles are broken into small particles. The broken particles fill the smaller voids, making the volume smaller. When the load increases, the broken particles are deformed again. The compaction process will go through one or more of the above-mentioned change processes that compact the pharmaceutical powder tightly together [2,10].

However, certain problems may arise during and after compaction, including looseness, capping [11,12,13], lamination [12,14], sticking [15,16], delayed disintegration [17,18], and differences in weight. Currently, the main studies are at the macro level, which makes it difficult to study particle behavior within the compaction process. The microscopic parameters of the compaction process are difficult to quantify. Therefore, it is crucial to explore the relationship between microstructural changes and find a suitable solution for the problems that arise during compaction [19]. Computer technology has enabled numerical technology to be associated with compaction and computer simulation, giving us microscopic information on powder particles during compacting.

Numerical simulation emerged in the middle of the 20th century, and it was first used to solve simple problems for one-dimensional meteorologically unstable radial and linear flows. With the development of computer technology and numerical simulation algorithms, numerical simulation has become a powerful tool for solving a wide range of micro-level problems [20,21]. It is helpful to provide an in-depth understanding of the compaction process [22,23,24], optimize process parameters [25,26], solve complex problems, and predict suitable formulation [25,27], in order to reduce material costs and improve product quality. Numerical simulation technology includes the discrete element method (DEM) [26,28], finite element method (FEM) [29,30], computational fluid dynamics (CFD) [31,32], population balance model (PBM) [33], and boundary element method (BEM) [34,35]. At present, these methods are used to solve various problems during compaction. Zheng et al. [28] used the DEM to study the die-filling phase, discussed the influence of stirrer design on filling performance, and determined the optimal design of the stirrer shape. Ohsaki et al. [36] used the FEM to describe the deformation behavior of tablets. Experiments were carried out to investigate the relationship between compression speed and plastic deformation. The plastic deformation of tablets was small at high compression speeds, and the load curves were consistent with the experimental data at different compaction speeds. The FEM helps to analyze the compaction phase. Dingeman et al. [25] used the FEM to simulate the process of multi-component pharmaceutical powders compaction. Experiments were conducted to predict the compaction curve and shear stress distribution of the mixture by the extract density-dependent Drucker–Prager Cap model parameters. This approach allows rapid exploration of powder formulations in early-phase development, providing information that is critical to the design of experiments, leading to cost savings. Widartiningsih et al. [37] used the CFD-DEM to investigate the effect of air on the powder flow behavior in the die-filling phase. This study should simulate the gas–solid flow behavior of the filling process under the invariance of the macroscopic properties of the particles, in order to optimize the die-filling process. This method can effectively simulate industrial filling systems. At present, numerical simulation methods have been applied to all operation units of tablet preparations, including drying, mixing, granulation, tableting, and coating [38]. However, it is considered a practical, effective, convenient, and resource-saving technique for generating crucial information and predicting compression results during compaction [20].

At present, detailed information on the various stages, including the compaction process, as well as the gradual transition from the macroscopic compaction stage to the internal microstructural change research stage, accompanied by the introduction of numerical simulation technology, is improving gradually. Previously, a review study was conducted that only focused on a single process and single numerical simulation method. However, there is a lack of numerical simulation studies of the entire compaction process, which is addressed in the present study. This study aims to analyze the existing literature and provide a comprehensive review of the application of numerical simulation during tablet compaction. The review includes the origin of numerical technology, the application of numerical simulation during the compaction, and the coupled application of multiple numerical simulation technologies. The difficulties of numerical simulation technology and the limitations of the current research are also discussed. Among these, the application of numerical simulation technology in various fields is the focus of this study, which will be introduced in this paper.

## 2. Application of a Single Numerical Simulation Technology for Compaction

The purpose of applying numerical technology is to deeply understand the parameters that cannot be determined in the actual operation process of each stage of the compaction process. It provides data support for accurate experimental results and optimization of experimental schemes. Currently, the commonly used numerical simulation methods are DEM, FEM, CFD, BEM, and PBM. However, only the DEM and FEM have been extensively studied for research on the compaction process, and the other numerical simulation methods have received less attention.

### 2.1. DEM

DEM is a numerical simulation technology, proposed by Cundall in 1971 that originates from molecular dynamics, initially used in the study of rock mechanics, and then gradually applied to the field of bulk materials and powders. The DEM is a computational method used to analyze the dynamics of material systems and is specifically designed to solve the problem of discontinuous media in bulk materials. Since bulk materials normally exhibit complex and mechanical motion behaviors, these behaviors are difficult to explain by existing medium theories. Therefore, the parametric modeling of bulk materials using the DEM provides information on material dynamics that is difficult to obtain from traditional experiments, such as the trajectory of material, the velocity of material at each moment of motion [39], and the stress distribution between particles [22,40]. It is also possible for the DEM to perform particle behavior analysis and simulation to solve the comprehensive problems of designing numerous particle motions, flows, energy distributions [41], and electromagnetic couplings [42], etc. With the rapid development of computers, elements within a DEM system can be analyzed millions of times by advanced computers, considerably increasing the scope of application of DEMs [43].

DEM is mainly used in the filling and compaction stages. In the die-filling stage, the powder flows into the die cavity via the feed frame, which impacts the total mass of the drug product, tablet strength, and content uniformity [26]. Physical tests have a limited understanding of particle-level interactions, but the DEM can describe particle-level details and provide insight into particle-level dynamics in the die-filling stage [26,44]. As a result, the DEM has become a popular research method. Current research has focused on the powder flow behavior in the feed frame [26], stirrer design [28,45], continuous production of pharmaceuticals [46], and the relationship between the process parameters and the filling performance [44]. Kumar et al. [26] investigated the effect of parameters, such as paddle speed and turret speed, on residence time, particle traveling distance, shear work and tablet quality on powder flow. The results revealed that high paddle speed significantly reduced the mean residence time, increased the shear work, and the distance traveled by the particles, and that it resulted in lower tablet quality. Excessive turret speeds can reduce particle residence time and shear work, thereby reducing the potential for excessive lubrication and material wear, which results in more homogeneous tablet mass. Zheng et al. [28] designed different types of stirrers within the feed frame, which significantly improved the filling performance of free-flowing powders. Mostafaei et al. [47] investigated the flow behavior of feed frames with different scale sizes. The results reveal that the mixing time and cohesion of the powder are related to the range of shear exposure, which affects the powder flowability. It is noted, in particular, that most of the DEM studies have focused on the die-filling process for rotary filling systems [26,48]. In the compaction stage, the DEM defines the position, velocity, and physical state of the particles in terms of individual particles [49], and focuses on the cohesive behavior of powders by introducing surface energy. However, the use of numerical simulation to study the compaction stage mainly focuses on the internal stresses, strains, and process parameters of compaction of the tablet. It is usually represented in the form of a mesh division and parameter cloud diagram. Therefore, the compaction process has been mainly studied using FEM methods, while compaction has been less studied using DEM. The main focus is on powder cohesion, including sticking behavior [50], cohesive powder cohesion behavior, etc. [49]. Table 1 summarizes the application of the DEM in the compaction process, mainly including the simulation process, the model, and the particle size, and describes the purpose, advantages, and disadvantages of the investigation.

**Figure 1:**
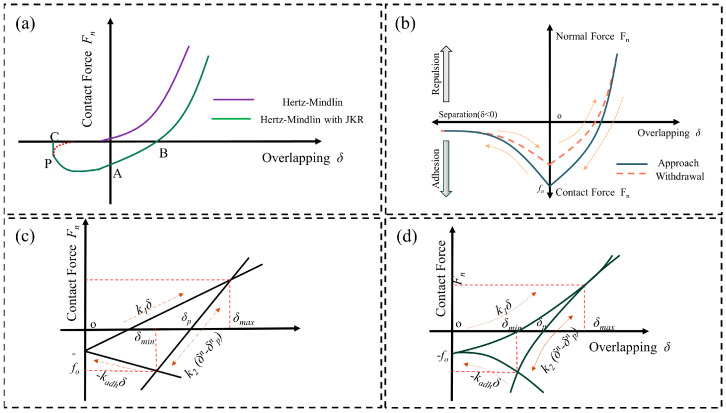
Schematic diagram of the DEM function. (**a**) The Hertz–Mindlin and the Hertz–Mindlin JKR cohesion model, and (**b**) force–separation curve inside the EEPA model (**c**,**d**) show linear and non-linear contact force–displacement function with the EEPA model, respectively.

In DEM, material–material and material–wall mutual contact is treated as a dynamic process, where each element is solved in an explicit numerical format [57]. Newton’s laws of motion are the basis of DEM contact models, and common models include the Hertz–Mindlin model (no slip), the Hertz–Mindlin and Johnson–Kendall–Roberts (JKR) model, the Edinburgh elastic–plastic adhesion model (EEPA), and the hysteresis spring model. The schematic diagram of DEMs is shown in Figure 1. During simulation of a particulate system using the DEM, the motion of each particle is governed by Newton’s second law, including translation and rotation motions [28]. The motion state of the material is shown in example 1, 2 of the equation. Table 2 summarizes the application of commonly used DEM contact models

(1) F=ma,

(2) M=Iθ,

where *F* is the net force applied, *m* is the mass of the particle, *a* is the acceleration of the particle, *M* is the net moment of force, *I* is the moment of inertia, and *θ* is the angular acceleration of the particle.

The Hertz–Mindlin model (no slip) originates from Hertz’s contact theory and Mindlin–Deresiewicz work, which is the basic contact model, applied to non-adhesive and incompressible material. When the particles are in contact with each other, the contact stresses generated between the particles are considered as face contact and are transformed into tangential and normal forces [58]. Figure 1a shows the relationship between contact force and overlapping in the Hertz–Mindlin model. The flow behavior of bulk powder was studied using the Hertz–Mindlin model (no slip) by Ketterhagen et al. [51]. Bulk powder with a particle size of 100 μm was represented by 2 mm non-rotating spheres, and other parameters were determined by applying a ring shear tester RST-XS. The experiment simulated the residence time distribution of powder flow in the feed system of a Fette tablet press. The aim was to better understand the effect of feeder design on die-filling performance. At different rotational speeds, the simulation of MCC and SiO_2_ mixtures showed to be consistent with the real flow behavior of the powder. The results reveal that the Hertz–Mindlin model can reasonably predict industrially relevant powder flow.

The Hertz–Mindlin with JKR model is based on the Hertz–Mindlin model (no slip) and introduces the Johnson–Kendall–Roberts cohesion contact model. Particles in this contact mode tend to adhere to each other (Figure 1a). Therefore, an additional force is required to separate them from the particles, which computes the additional work required to physically separate the particles to break the adhesion contact. The model commonly applies to adhesive systems but can also be used for incompressible materials [59]. Kumar et al. [26] used the Hertz–Mindlin model with the JKR model to investigate the flow behavior of powder in the feed frame of a tablet press. The powder flow behavior of particles with a size distribution of 0.55–1.30 mm under three paddle rotational speeds of 20, 40, 60, and 80 rpm with turret speeds of 20, 40, 60, and 80 rpm, respectively, was investigated using distribution paddles, dosing paddles, and filling paddles. Lower paddle speeds and turret speeds resulted in longer particle residence times and shorter distances traveled. Higher paddle speeds and lower turret speeds resulted in higher shear work. The distribution paddle is subjected to about five times the torque of the dosing paddle and twice the torque of the filling paddle. Additionally, increasing the paddle speed helps to homogenize the filling, but decreases the filling weight. The results demonstrated that the Hertz–Mindlin model with JKR model predicts the powder flow behavior and tablet filling mass well.

EEPA is a non-linear hysteresis spring model for explaining history dependence and the main characteristic behavior of adhesive material [60]. The model captures the historical dependence and the main characteristic behavior of cohesive particles. Figure 1b shows the relationship between the normal force and overlapping in the EEPA model [61]. The dashed arrows in the figure indicate the process of overlapping (δ > 0) and separation (δ < 0) between the particles after the contact force or normal force (F_n_) is applied. The solid blue line shows the particles in approach and the dotted red line shows the process of withdrawal between particles. The model consists of a non-linear hysteretic spring model that considers elastic–plastic contact deformation and adhesion components as a function of plastic contact deformation, which commonly applies to adhesive systems and compressible materials. Figure 1c,d are the schematic diagrams of the linear and non-linear modes of the EEPA contact spring in the normal direction, respectively [62,63]. The model provides an ever-present adhesive force and other normal forces by applying a constant pull-off force, including the loading spring, the unloading and reloading spring, and the adhesive spring phases [64]. It corresponds to the approach of two particles to each other and their elastic deformation, the plastic deformation due to the switching of the contact spring during unloading of the two particles to the unloading and reloading spring, and the contact between the particles during particle separation, respectively [65]. Benque et al. [46] simulated an industrial-scale feeder frame mixing process using the EEPA model by the DEM. Two APIs and an excipient with a particle size of 4.5 mm were mixed in a feeder with a 0.4 m long blade, and a 45° angle of rotation axis was added as a secondary baffle. The direction of convection mixing is changed by rotating the feed frame by 90° after 10 min of the mixing process. It accelerated the mixing speed and reduced the powder segregation. The trend of numerical simulation prediction is generally consistent with the experimental data, indicating that the use of the EEPA can better assess and reduce the risk of segregation of pharmaceutical powders.

The hysteretic spring model is a contact model that allows elastic deformation behavior to enter into the equations of contact mechanics. It exhibits elasticity when the particle reaches a predetermined certain stress. It is mainly applied to non-adhesion systems, but also to compressible materials. Once stress exceeds a certain value, the elastic deformation of the particles is transformed into plastic deformation. The results revealed that a large overlap between the particles occurs without excessive force, thus reflecting the properties of compressed materials [66]. Xu et al. [67] used the hysteretic spring model to investigate the segregation phenomenon of die filling in a single-punch tablet press. The die diameter was 2 mm, depth was 6 mm, particle size was 4.5 to 735 μm, and the feed frame was filled at a feed rate of 45 mm/s. Owing to percolation, the smaller particles in the interstices of larger particles sink to the bottom of the feeder, resulting in particle stratification in the vertical direction. The greater the coefficient of friction (COF = 0.8), the greater the degree of particle segregation (SI = 0.07). As the charged mass in the feed frame increases, particle packing restricts vertical movement, and the degree of segregation decreases significantly. The results revealed that the application of the hysteretic spring model predicts the segregation in the powder flow behavior well.

### 2.2. FEM

FEM is generally a numerical simulation technology for solving approximate solutions to boundary value problems using partial differential equations (PDEs) [38], which is applied to the analysis of complex mechanical problems, model powder compaction, predict tablet behavior [68], etc. It was first introduced by Clough in 1960 when he analyzed the problem of plane stresses in the elastic limit range. It discretizes the continuous domain into finite units and expresses the physical behavior of each unit mathematically. The equations of each unit are related and solved to obtain a mathematical expression of the whole system [69]. The FEM is a fast, efficient, and reliable computational method for reducing experimental costs and predicting product quality. With the continuous advancement of computer technology, many computer-based FEM software have emerged, such as ANSYS, ABAQUS, and SciFEA [69].

For a deeper understanding of the compaction behavior of tablets, the FEM has been applied to pharmaceutics to provide parameters that could not be obtained through experimental testing, including tablet failure mechanisms during compaction and ejection [12], temperature evolution [70], internal stresses and density distributions within the tablet [71], and the effect of punch shape with other mechanical properties [72,73]. The FEM is based on the powder compaction models and defines the contact model according to the different properties of the powder, mainly focusing on solving the displacement, strain, stress and internal deformation of each element [74]. Currently, the experiment is modeled for different shapes of tablets to study the tensile strength of biconvex and capsule-type tablets [73]. The FEM was used to investigate the internal and residual stress distribution within tablets, predict the capping trend of convex tablets, and identify the optimal parameter combination of geometric parameters and friction coefficients [75]. Mazel et al. [12] investigated the tensile stress portion of tablets by changing the unloading conditions. The results showed that maintaining a smaller pressure on the side of the tablet can reduce the risk of tablet defects with high capping or lamination tendency. Therefore, powder compaction is modeled by the FEM to address the compaction trouble at the micro level, thus saving materials, time, and other resources in the pharmaceutical industry [71]. Table 3 summarizes the application of the FEM in the compaction process, mainly including the simulation process, the model and the material, and describes the purpose, advantages, and disadvantages of the method.

In order to investigate the microstructure and performance of materials, the FEM system combined with the constitutive model is needed to describe the compaction process and mechanical strength of materials. The parameters of these constitutive models are input into the FEM system for analysis to obtain the information inside the material. Common constitutive models include linear elasticity, linear viscoelasticity, elastoplastic, and Drucker–Prager Cap (DPC) models.

The linear elastic model is the basic constitutive model. When the applied loading is 0, the material compacted into a low-porosity powder body undergoes elastic deformation. The elastic behavior of most of the materials is not homogeneous, and to simplify the process, the model assumed that each material is isotropic with linear elastic behavior [79]. Linear elastic modeling is mainly applied to characterize the elastic parameters of isotropic or anisotropic materials by pulsed excitation techniques in combination with the FEM [11,76,79]. The model does not consider the cohesive behavior of the material. Mazel et al. [79] used pulsed excitation technology combined with the FEM to model the powder body to assess the elastic parameters of the tablets. Four classical excipients (Alac, Mlac, MCC, ACP) mixed with MgSt were modeled and compacted in FEM software using a linear elasticity constitutive model. The pulsed excitation technique is based on the detection of the intrinsic resonance frequency of the solid. The Young’s modulus and Poisson’s ratio of the tablets were determined from the two lower vibration frequencies of the tablets. It was found that the deviation of Young’s modulus was less than 0.06 GPa and Poisson’s ratio was less than 0.01 for each batch of tablets, which accurately assessed the elastic parameters of the tablets. The linear elasticity model can be applied in the tablet compaction process that assumes isotropic materials.

The linear viscoelasticity model is a constitutive model describing composite materials, which is based on the linear elasticity model [80]. It extends to the linear viscoelastic models in the Laplace transformed domain. Elastoplastic models are mainly used in materials with elastic and plastic properties, which are generally divided into classical elastoplastic models and reinforced elastoplastic models. Desbois et al. [68] used the FEM in association with a viscoelastic constitutive model to investigate the relationship between powder compaction properties and strain rate sensitivity (SRS), which was expressed in terms of the laws of linear elasticity and viscoelasticity of the Prony series. The SRS is affected by friction, viscoelasticity, viscoplasticity, and air trapping. Microcrystalline cellulose (MCC), starch, lactose hydrate (GLac), and anhydrous calcium phosphate (ACP) are used as compaction materials. The results showed that ACP had the largest porosity (26.2–32.4%) under pressure, while starch had the smallest porosity (6.4–10.5%) under pressure. The parameters of G, K, T, E, and v of each material were characterized. The results show that GLac and ACP have very limited viscoelasticity, and MCC is more viscoelastic than starch. The viscoelasticity model is well applied to cohesive pharmaceutical powders.

**Figure 2:**
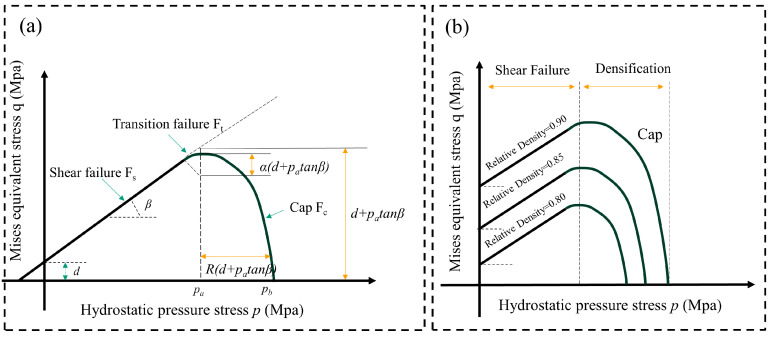
Schematic diagram of the FEM function. (**a**) The Drucker–Prager Cap model and its parameters, and (**b**) the Drucker–Prager Cap model with different relative densities.

The DPC is the most commonly used constitutive equation applied to the compaction process [81]. It is based on the Mohr–Coulomb model, and it revised the von Mises yield criterion and added additional terms to the von Mises equation. The model considers the angle of internal friction of the material and the Cap model at high hydrostatic pressure subjected to constraints [82]. The model assumes that the material is isotropic, and the compaction process of the material is analyzed by the DPC model. The model considers volume expansion due to the yield but does not consider the effect of temperature changes. The DPC model describes the yield surface track of the tablet, which comprised two faces, as shown in Figure 2a. The first one is the shear failure line, and the other one is the Cap line. These surfaces are described in the space of hydrostatic pressure p and von Mises stress q. The hydrostatic pressure is the stress that is only related to a change in the volume of an object without changing its shape. The von Mises stress is a measure of shear stress that is associated with changes in the shape of an object without changes in volume [71,83]. However, complex compaction processes are difficult to represent accurately with DPC models. For example, there is a hardening surface in the compaction process [82]; the parameters of the DPC model are related to the relative density (Figure 2b) [71]. The variation in the yield surface with relative density depends on the compaction process subjected to different compressive forces, the relative density affects the shape of the yield surface [84]. Therefore, the tablet compaction behavior can be simulated well by using a modified density-dependent Drucker–Prager Cap model [25]. LaMarche et al. [71] used the FEM combined with the DPC constitutive model to simulate the relationship between density and stress distribution in compaction. The model is commonly used in the pharmaceutical industry to determine the utilized-early formulation development process and avoid manufacturing and scale-up risks. This research summarized the DPC parameters of 60 kinds of materials, including microcrystalline cellulose, lactose, anhydrous lactose, mannitol from different suppliers, and API materials in different proportions at different project stages. By collecting the general and particular behaviors of these formulations, the results showed that the elastic modulus (E), Poisson’s ratio (v), cohesion (d), friction angle (β), and eccentricity (R) of the cap surface are all related to the relative density. Based on the analysis of the range values of the parameters, the trend between the excipient, the API blend, and the final blend can be determined. The parametric distribution shows that most of the materials vary slightly from the median and only a few materials parameters are distributed at extreme values (E = 1–14 GPa, v = 0.23–0.37, d = 0.2–15 MPa, β = 65–72°, R = 0.65–1.15, hydrostatic stress and the equivalent stress ratio (q/p) = 0.2–0.675, hydrostatic yield stress (P_b_) = 40–140 MPa). The results demonstrated that understanding the classical parameter range of the material, as well as the risk types of extreme values, can help to predict and effectively avoid potential material compaction issues. The DPC model predicts the compaction process, which provides a visual basis for exploring new formulations, reducing the risks, and saving time and economic costs [24,25].

### 2.3. BEM

The boundary element method (BEM) is a numerical simulation method widely used in engineering, physics, and acoustics [85,86,87]. In 1967, Rizzo proposed the idea that the boundary integral equation of elasticity is discrete according to the boundary, which is the beginning of the boundary element method. BEM is based on boundary integral equations. The differential equation in the region is transformed into the integral equation on the boundary of the region, whose undetermined coefficients are determined by the satisfied boundary conditions. Previous studies have found that the biggest problem in developing BEM is the difficulty in solving its system of linear algebraic equations. To solve this problem, current research is introducing fast computation into the BEM, with the potential for the calculation of complex engineering problems [34]. In contrast to traditional methods such as the FEM, the BEM focuses primarily on the boundaries of the problem. The boundary is divided into discrete elements, thus, greatly reducing the number of degrees of freedom required, while achieving the same level of precision, by establishing integral equations on the boundary to describe physical phenomena [86]. The BEM has effectively solved the unknowns and the large width of the stiffness matrix due to the dense division of stress concentration regions [88]. The BEM has higher accuracy compared to the FEM, and it is easier to analyze the complex behavior of the boundary with less discrete error. The BEM is more convenient for solving infinite domain problems. In solving problems with infinite or semi-infinite domains, it is necessary to introduce the infinite domain BEM to describe the infinite domain [89].

The BEM is mainly divided into two kinds of solving equations: direct boundary element equation and indirect boundary element equation [90]. The two equations are developed on the basis of Green’s function. The direct boundary element equation is derived and discretized using Green’s function and Laplace’s equation. The unknown quantity of the equation is the boundary quantity directly related to the function to be evaluated by the Laplace equation, which has a clear physical meaning. The indirect boundary element method introduces some virtual density function to the boundary to construct the boundary integral equation through the potential theory. This equation is related to the density function, but the density function is not directly related to the Laplace equation. Therefore, it is called the indirect boundary element equation. However, there is no precedence for the application of BEM for compaction.

### 2.4. CFD

Computational Fluid Dynamics (CFD) is a discipline that uses digital computers to quantitatively predict fluid motion based on the laws of conservation of mass, momentum, and energy that govern fluid motion [91]. Recently, CFD has become an important methodology in materials, biochemical engineering, and pharmaceuticals. CFD can predict key information about a variety of processes—mixing properties of materials, shear stress variations, temperature variations, and so on. This knowledge helps to further the understanding of flow and heat transfer processes in various applications [92].

CFD is a tool for experimental design and numerical simulation and has several distinct advantages, including cost saving, rapid computation, and the ability to understand information about the entire watershed with relevant variables. Simple parameter changes can be made to optimize the experimental design in a large computational domain [91]. CFD is mainly used in filling processes, including the flow of fluids and the interaction between air and material flows [93,94], etc. According to their motion behavior, fluid properties are classified into continuous incompressible fluids and viscous incompressible fluids. The continuous incompressible fluids are mainly molten metal, which are beyond the scope of this review. The motion state of continuous incompressible fluids is governed by continuity, momentum, energy, heat flow, and enthalpy constitutive equations [95]. The continuity equation, momentum equation, and energy equation describe the volume of fluid. The heat flow equation and enthalpy constitutive equation describe heat transfer and solidification [96]. Viscous fluid behavior includes friction, heat conduction and mass diffusion. The viscous incompressible fluids are governed by the Navier–Stokes equation [97]. However, CFD is rarely used solely to simulate the flow of materials. Because pharmaceutical powders are mainly in the form of solids, the continuity and energy equations of CFD are applied in combination with the DEM to investigate the flow behavior of multiphase materials.

### 2.5. PBM

The population balance model (PBM) is one of the essential methods for describing the evolution of properties on the scale of granular processes. The population balance equation (PBE) is an important integral differential equation in the PBM. It uses a mathematical kernel to represent the physical properties of the process [33]. The PBM is a widely used method to describe crystallization processes. The method requires consideration of the particle size evolution characteristics of discrete phase particles, including the particle size distribution of solid particles, bubbles, and droplets. Distributions can be accompanied by a variety of reactions and time-dependent transfer phenomena. Changes in particle size include relationships such as nucleation, growth, dispersion, dissolution, incorporation, and fragmentation [98]. Ashley et al. [99] quantified the granule growth rates of different formulations, used the PBM with a statistical model to represent the processes of granulation, milling and compaction, and predicted powder and tablet properties. The PBM is commonly used in investigations related to various aspects of the crystallization of particles, granulation, emulsification and separation, and fluidized beds. Presently, the use of the PBM method to simulate the compaction process is not being investigated [100].

## 3. Application of Combined Numerical Techniques for Compaction

A single numerical technique makes it difficult to meet the current research needs. For example, the DEM still has several drawbacks. Computational efficiency is too low when the number of materials reaches a certain order of magnitude. In the FEM, the phenomena such as interparticle contact, rolling, sliding, cohesion, and friction between materials cannot be characterized [101]. However, researchers coupled numerical techniques to break through the current troubles.

### 3.1. DEM-FEM

The DEM usually simulates the motion behavior of granular materials by applying external forces or material–material forces and torques on individual materials. However, idealized assumptions can apply to some materials, and these assumptions limit the applicability of the DEM. The DEM usually assumes that particle interactions are rigid or elastic, but the DEM is unable to accurately account for collisions of these materials as viscoelastic and elastoplastic, etc. [102]. The DEM simplifies the algorithm of particle contact, which affects the accuracy of density prediction and the observation of particle deformation. It is not suitable for compaction simulation [103]. The FEM can analyze the behavior of continuous materials and structures. It accurately simulates the process of plastic deformation of material under loading [104]. The FEM characterizes the mechanical behavior, density distribution, and displacement changes in the material through the constitutive equation of the material. However, the material motion behavior cannot be understood [101]. Therefore, a method to solve the challenges associated with the FEM and the DEM is the use of the multiparticle discrete element method (MPFEM) [105], which combines the characteristics of the FEM and DEM. The MPFEM models the powder as a discrete body and contains a full mesh of units, which allows properties such as cohesion, support reaction force, von Mises stress, etc., to be achieved at the particle scale during simulation calculations [105,106]. This method generates random material packing by DEM software, and the deformation of materials was characterized by the contact overlap between materials [107]. However, the DEM cannot accurately describe the large-scale deformation when the relative density is greater than 0.85 [108]. The FEM takes into account the large deformation and discrete characteristics of materials through display dynamics analysis, and analyzes the microscopic parameters such as stress–strain between particles through mesh division. Therefore, this method is accurate for the behavioral characterization of powder compaction that allows effective observation of the deformation, adhesion, and cohesion behavior between particles [101]. Firstly, the properties are defined in the particle factory of the DEM and imported within the FEM software to generate particles to simulate the filling process and then loads applied to the model are used to simulate the compaction process. The MPFEM has greatly contributed to the understanding of the compaction process at the microscopic level. However, there are still limitations of this method, including the fact that the morphology of all powders is modeled as spherical, making it difficult to characterize the realistic morphology of the powders. Since the number of realistic models are far beyond the computational range of the computer, the computational cost needs to be fully considered in the simulation.

Currently, it is mainly applied to the compaction and isothermal hydrostatic pressing of polymetallic powders [109], where the Johnson–Cook model has been used to analyze the strain rate, hardening law, and temperature softening effects of material [110], etc. However, the MPFEM is a suitable method, and is still rarely applied for the simulation of compaction behavior. For example, the DEM is applied to model irregular cohesive particles, and a new model is proposed based on the normal contact force. The compaction behavior is simulated by the FEM, and the results of the simulation are basically consistent with the experimental results [111]. Ku et al. [101] used the MPFEM to model cohesive particles to simulate the mechanical response of cohesive and plastically deformed particles during compaction. A new understanding of the elastic, plastic, yield, and damage hysteresis behavior of powders was provided.

### 3.2. DEM-CFD

Pharmaceutical powders are usually subjected to the process of gravity filling, suction filling, gravity mixing, fluidization transport, and drying granulation, etc. As the particles are subjected to various processes, the contact forces of particles colliding with each other are relevant to the motion and behavior of the particles [112]. DEM-CFD coupling can be used to study the microscopic behavior of particles and the interactions between particles. The DEM models the motion of particles, while CFD is used to solve for the motion of gases or fluids, which provides a more realistic understanding of the relationship between the motion of the continuous and discrete phases [113]. The DEM-CFD is mainly applied to fill the stage, the DEM to model the movement of pharmaceutical powders, and CFD to investigate the flow behavior of powders mainly on the effect of airflow (vacuum state, air, etc.) [114], and charged particles [113]. Table 4 summarizes the application of the DEM-CFD in the compaction process, and introduces the simulation stages, contact model, materials, research purpose, advantages and disadvantages, etc.

Yao et al. [93] applied a coupled DEM-CFD approach to the whole die-filling process and modeled the gas–solid flow in the boundary system. Experimental calculations of particle–air interactions were performed to verify the relationship between actual experiments and simulations. The experiment used NONPAREIL-108 particles with a diameter of 200 μm to simulate the die-filling process. It was shown that the filling rate with a shoe velocity of 0.10 m/s is less than that of 0.047 m/s. The slower the shoe speed, the higher the fill rate. In simulating the airflow in the shoe, the highest air flow rate occurs during the particle die-filling process and close to the die. In the early stages, the air–fluid first flows downwards with the particles, and once in contact with the particles or the wall, there is a counter-current effect leading to a reduction in the accumulation of particles. The CFD-DEM coupling accurately simulates the material filling process affected by air between the solid and gas phases.

### 3.3. Other Simulation Method

The combined use of multiple numerical simulation technologies is a common method for simulation. Due to the limitations of some models, it is impossible to design comprehensively and analyze the system, failing to achieve the desired situation. Therefore, other numerical simulation technologies or methods are required for coupling. Molecular dynamics (MD), which can represent the particle motion trajectories and microscopic details of condensed systems, is used [117]. Smooth particle hydrodynamics (SPH), a method of interactivity between particles via a smooth function, is commonly used to solve problems with a large degree of deformation [118]. The bonding particle model (BPM) is a model that is suitable for particles that have adhesive properties between them, which will model the fracture and bonding behavior [119]. The multi-contact discrete element method (MC-DEM) is a method for solving the problem of non-spherical particles. It combines the bonded multi-sphere method (BMS), which incorporates intragranular bonds between particles, and the conventional multi-sphere method (CMS), which allows the overlap between particles to form rigid bodies [120]. Compaction is currently only studied in depth by the Johnson model or MC methods [120,121]. However, multi-scale problems can be solved by applying coupled methods in other fields. Table 5 summarizes the application of other simulation technology in the compaction process with other fields, and introduces the simulation stages, contact model, materials, research purpose, advantages and disadvantages, etc.

Khoei et al. [122] investigated the compaction process of metal nano-powders by applying the FEM-MD multiscale technique. MD as a potential data method to study the compaction process can effectively simulate uniaxial compaction behavior [126]. The nano-powder’s body was assumed to form a continuum, which was modeled using non-linear finite elements. Atomic RVEs were modeled using the MD method to catch the plastic deformation of the nanoparticles during the compaction process. To demonstrate the mechanical behavior of RVEs under different loading paths, 64 Au nanoparticles with direct 50 Å were generated. Different compaction velocities (1, 0.1, 0.01, 0.001 Å/ps) were applied to perform compaction experiments. It was shown that the highest dislocation length and number of dislocations of the particles were 15,000 Å and 1200, respectively. When the compaction speed was 0.1 Å/ps, the highest content of the atomic number of amorphous structures on the surface (70%) was observed. However, the FCC structure has the lowest percentage of content (23%). The results demonstrated that the friction of the walls can be divided into three types of zones: rearrangement, plastic deformation, and cold working of nano-powders. The friction coefficients are essentially constant in the first and third zones, while in the second zone, the friction coefficient tends to decrease. Thus, this continuous multiscale approach analyses the compaction effect on nanoparticles. The results showed that the FEM-MD method is suitable for the compaction process of nano-powders, revealing the discrete nature of nano-powder and verifying the accuracy of the multi-scale analysis results. MD is presented as a highly promising technique that can be applied to the tablet compaction process. It has been shown that thermal analysis and MD are used to study the adhesion behavior of the tablet compaction process [127].

## 4. Challenges and Difficulties in Numerical Simulation of Compaction

### 4.1. Calibration of Powder Parameters

In recent years, a large number of experiments have been carried out to calibrate the parameters of powder, which have been validated by various experiments. However, there is a lack of research on the parameter calibration of powders because of their complex properties. The main difficulties are discussed below.

Powders are micronized particles with complex physicochemical properties that are difficult to quantify, as reflected in the parameters measured. Poisson’s ratio is the ratio of transverse and longitudinal deformation produced by the powder. The powder particles are microscopic, and Poisson’s ratio of individual particles is not measured in the actual calibration process. Therefore, Poisson’s ratio is estimated using the overall method, which is inaccurate. It is difficult to quantify other parameters of powders, such as elastic modulus, shear modulus, particle-to-particle coefficient of rolling friction, and coefficient of static friction, etc. The elastic modulus is the ratio of the stress–strain curve generated during elastic deformation, and the shear modulus is the ratio of the shear stress to strain over the strain range. Both parameters are easy to calibrate for materials with a large mass volume, while they are difficult to calibrate for powders. The reason is that it is not possible to accurately define the range of elastic deformation and plastic deformation of powder under a certain pressure. According to the microelement method and differentiation, it is necessary to estimate the elastic modulus of powder by the transition surface between elastic deformation and plastic deformation. The most critical issue is maintaining a homogeneous powder surface for the calibration of particle-to-particle coefficients of rolling dynamics and static friction. When the powders are in contact with each other, the sliding distance and critical motion parameters of the powders need to be accurately and stably obtained. During the calibration of powder properties, external conditions such as temperature and humidity have an impact on the properties of the powders.

In the process of importing the parameters of powders into the DEM, FEM, etc., the numerical parameters are set differently due to the different nature of each powder. The software produces up to a million or even ten million or more orders of magnitude of powdered particles. Due to the enormous number of calculations, it is difficult for general computers to perform fast calculations, which need an advanced computer or the combined use of multiple CPU/GPUs to perform the calculations. Powder calibration using numerical simulation technology saves a lot of experimental costs but requires much time and computational resources.

For the whole powder compaction process, it is necessary to find a suitable constitutive model to describe the behavior of material densification. The Drucker–Prager Cap model, which is often used to examine the state of change within the compaction, introduces the Cap surface. However, the parameters of the DPC model need to be accurately defined, including the eccentricity parameter (R), friction angle (β), evolutionary parameter (P_a_), and yield stress–plastic strain relationship, etc. The DPC model can be used for compaction in a wide range of applications. It is difficult to accurately define the density-dependent DPC parameters with the finite element software. It is necessary to define the DPC parameters related to relative density through subroutine development and programming design using computer language. In the future, when the composition of material changes from a single material to multiple materials, its internal parameters will be difficult to define. The current study only defines the DPC parameters for a mixture of two materials. When the material composition is complex, the compaction properties of these materials are difficult to predict.

After the calibration of the powder, the characterization of the calibration results needs to be compared with the actual results to judge the authenticity of the calibration parameters. In the subsequent numerical simulation of compaction experiments, the question of how to use the calibrated parameters to complete the compaction process and how to practically apply the simulation experiments of these calibrated parameters to the actual production process is crucial.

### 4.2. Difficulties in Numerical Simulation

Numerical simulation technologies are commonly used to solve the issues during compaction. However, it still has some deficiencies. The DEM is difficult to calculate quickly for a large number of particles and is unable to accurately calibrate the particle parameters. Currently, most of the studies have simplified the shape of the material in the simulation. The characterization of the true shape of the material by microscopy technology may cause an increase in the number of computational challenges. As the most popular method of the compaction process, the FEM is mainly associated with constitutive equations to simulate the compaction stage. It is difficult to find a suitable constitutive model and calibrate its parameters in the FEM in order to predict the actual situation in the compaction stage. By expanding the new constitutive model or modifying the constitutive model (for temperature and density), the compactibility of materials can be better characterized. The coupling of the DEM and the FEM is mainly used in the die-filling and compaction stages. At present, the MPFEM is mainly used to solve 2D problems. However, a large number of computational challenges need to be overcome for complex 3D problems. The 2D problem consists of a flat FEM simulation of the compaction process, in which the powder is treated as a continuum. The 3D problem consists of the die-filling phase using the DEM, the compaction process of the MPFEM, etc. The 3D problem simulation has a number of potential limitations, including excessive computational effort, difficulty in realizing the true particle size and morphology of the powder, and ignoring the natural packing behavior of the loose powders, which lead to issues in the simulation. It may be solved by other numerical simulation methods or meshless numerical simulation technologies. CFD mainly describes the flow behavior of fluid materials and is commonly used in the filling stage. The combination of CFD and the DEM can describe the motion state of multi-phase materials, such as gas, liquid, and solid phases. However, it is difficult to accurately define the interaction relationship between different phases. Due to the unknown of the contact interaction, the shape of the contact phase and the material flow may be irregular, which cannot be quantified for the cohesion performance of the material or the computation of the model. It can be processed by the co-acceleration method of multicore CPU and GPU, which has great potential for the DEM-CFD or other numerical simulation methods. The BEM is rarely used in the compaction process, because it mainly calculates the boundary conditions of materials and cannot obtain the internal material parameters. The BEM can indirectly express the stress–strain, deformation, fracture, and additional properties of materials by combining them with other numerical simulation technology, which provides new applications. The BEM can indirectly express the stress–strain, deformation, fracture, and other properties of materials by combining them with other numerical simulation technology, which is useful for providing news applications. For the coupling methods in other fields, it is hoped that they will be learned and applied to explore some new methods for large-scale compaction problems, including molecular dynamics and the lattice Boltzmann method.

## 5. Conclusions

In this paper, a relative review of the literature on the compaction process has been compiled using numerical simulation technology as a tool for studying the process of compaction. First, the theoretical basis for the compaction process is reviewed, including the principles of die filling, compaction, ejection, classification of each stage, and current trouble-shooting techniques. Subsequently, the application of numerical technology (DEM, FEM, BEM, CFD, DEM-FEM, CFD-DEM, etc.) in the compaction process is introduced, including the origin of each numerical simulation technology, numerical simulation model, and application scope. For the existing studies, the material properties, numerical models, research purposes, advantages and disadvantages, etc., are summarized. Finally, the paper discussed the difficulties associated with the calibration of material parameters in the compaction process and the limitations of current numerical techniques.

A series of conclusions can emerge from the in-depth observation of numerical simulation technologies in the compaction process, which also shows the direction and future trends of the techniques that are worthy of in-depth study in the present research context. Currently, it is necessary to develop a set of appropriate legislation for certain numerical simulation technologies and regulate the research of numerical simulation techniques. It has been noticed that appropriate legislation needs to have at least the following points. Firstly, with regard to regulations for compaction simulations, a clear set of rules on the scaling coefficient of particles needs to be developed. Currently, optimization of the experimental design leads to differences at different levels and should be harmonized between compaction simulation levels. Secondly, as the current powder morphology is irregular, the simulation optimization process treats the particles as single spherical particles, which can seriously affect the simulation results. Therefore, how do we go about regulating the optimized morphology of the particles? For example, what roundness can be considered as a single spherical particle, and what morphology can be considered as a non-spherical particle? Finally, there is a large difference between the packing of particles and the numerically simulated die filling of particles. How do we differentiate between the two and set a modifying coefficient to reduce the simulated behavior from the real experimental result accuracy? A further in-depth understanding of the relationship and differences between numerical simulation technologies and actual experiments is needed. By reviewing the literature of the last few years, it has been observed that most of the experiment mechanisms have been investigated, and that it is possible to try to summarize them. It facilitates the further discovery of unexplored coupling methods. The application of three or more numerical simulation technologies can better assist in understanding problems on a deeper scale, contributing to the development of future research. There is still a big gap between numerical simulation and actual experiments, and it is difficult to accurately estimate the conditions and variations in actual experiments. However, numerical simulation technology still plays a good role in guiding the trend of future research and will continue to deepen the research of numerical techniques in the future.

Numerical simulation technologies have been used to analyze the compaction process and successfully predict information about the material behavior of the compaction process under actual experimental conditions. Numerical simulation methods combined with experiments are widely used in various fields and have proven to be computationally fast, material-saving, practical, and efficient research methods for specifying crucial information about complex physical phenomena in compaction processes; for example, the segregation process of powder in die filling, the degree of uniformity of die filling, the stress–strain distribution of the compaction process, the fracture mechanism of tablets under loading, the compaction mechanism of dynamic magnetic compaction processes, and the residual stress suffered by the decompression process. In recent years, compaction processes and numerical simulation technology have been highly emphasized in various industries. In the future, numerical simulation technology will become increasingly attractive to researchers in the industrial sector owing to the development of new technologies.

## Figures and Tables

**Table 1:** Numerical application of the DEM during the compaction process.

Process	Numerical Method	Model	Material	Purpose	Advantages	Disadvantages	References
Die filling	DEM	Hertz–Mindlin with JKR model	Radius (D = 0.55–1.30 mm)	Investigation of powder flow in a three-chamber feed frame under different operating conditions	Particular attention was paid to the influence of the feed frame on the powder flow behavior	The tablet punch dosage phenomenon has not been simulated	[26]
Die filling	DEM	Hertz–Mindlin	Radius (D = 100 μm)	Investigation of the flow behavior of bulk powders of smaller particle sizes	Use of non-rotating spherical particles to represent fine powders	Lack of quantitative analysis of fines flow behavior	[51]
Die filling	DEM	Linear viscoelastic spring dashpot model	Radius (D = 200–800 μm)	Investigation of the effect of forced feeders on filling performance	Four stirrer designs are proposed to investigate the powder flow behavior	No studies on the effect of stirrers on the flowability of cohesive powders have been conducted	[28]
Die filling	DEM	Linear viscoelastic spring dashpot model	Radius (D = 200–800 μm)	Investigation of the die-filling process with a linear feeding system	Studies on the effect of a stirrer in the feed frame on filling performance	Influence of powder cohesion on filling properties not considered	[52]
Die filling	DEM	EEPA model	D_10_ = 24 μm, D_50_ = 231 μm, D_90_ = 1104 μm	Investigation of the distribution of residence time of the material in the tablet press	Residence time distributions of tracers were collected using optical measurements	Lack of research on material cohesion and rotational speed	[53]
Compaction	DEM	Elastoplastic normal contact model	Radius (D = 70 μm)	Investigation of sticking phenomena using the DEM	Studied the phenomena of particle cohesion and adhesion	Lack of cohesion studies on multi-materials	[50]
Compaction	DEM	Hertz–Mindlin with JKR model	Radius (D = 0.44–1.77 μm)	Investigation of the behavior of powder processing and handling properties	Studies of powder mixing, compression, segregation, cohesion problems, etc.	Wet granulation process produces complex cohesive properties	[49]
Compaction	DEM	Hertz–Mindlin model with JKR model	Loose powder bed (D = 5–65 μm)	Investigation of compactor simulation of powder bed densification process	Understanding the mechanism of action of the compaction process	No research on the roughness of the powder layer	[54]
Die filling	DEM	Hertz–Mindlin	Coarse and fine particles (D_c_ = 3.9 mm, D_f_ = 1.95 mm)	Investigation of the effect of feed shoe design on segregation	Reducing segregation in eccentric tablet presses	Lack of feed shoe to die cavity process	[55]
Die filling	DEM	MEPA model/linear viscoelastic spring dashpot model	Radius (D = 350 ± 50 μm)	Investigation of the filling behavior of different types of feed frames	Studied the flow behavior of free-flowing and cohesive powders	Lack of optimization for different paddle shapes	[48]
Die filling	DEM	Hertz–Mindlin with JKR model	Radius (D = 1129–2800 μm)	Investigation of the effect of paddle shape on tablet quality	Studies on the powder flow behavior of three paddles of different shapes	Computing capability constraints and excessive simulation time	[56]
Die filling	DEM	Hertz–Mindlin model	Radius (D = 0.6 mm)	Investigation of powder flow in a multi-granular approach	Improving simulation speed with MCG technology	Limitations of the MCG method remain	[45]
Die filling	DEM	Linear EEPA model.	Radius (D = 4.5 mm)	Investigation of an industrial-scale pharmaceutical powder die-filling stage	Using different numerical tests to reduce axial segregation tendencies	Higher cost of equipment modification	[46]

**Table 2:** Application and characteristics of the DEM.

Model	Applications	Characteristics
The Hertz–Mindlin model	Elastic collision	Suitable for most materials
The Hertz–Mindlin model with JKR	Viscous–elastic collision	Suitable for moisture-containing, viscous materials
The hysteretic spring model	Elastic–plastic collision	Suitable for materials with plastic deformation under stress
The Edinburgh elastic–plastic adhesion model	Viscous–elastic–plastic collision	Catch the cohesion of the collision process
The linear spring model	Elastic–plastic collision	Suitable for linear stress–strain relationships

**Table 3:** Numerical application of the FEM in the whole compaction process.

Process	Numerical Method	Model	Material	Purpose	Advantages	Disadvantages	References
Compaction	FEM	Drucker–Prager Cap model	Various MCC *, lactose, mannitol, PVP *, and pharmaceutical powders	Investigation of the distribution of density and stress in compacts	Studied the various excipients and pharmaceutical DPC parameters on compaction	Subroutine development is more complicated	[71]
Compaction	FEM	Linear elastic model	Alac *, SD-Lac *, ACP *, GLac *, IsM *	Investigation of the process of tablet compression failure	Using the cohesive zone model to explain tablet failures	No research on the tensile strength of the mixture	[76]
Compaction	FEM	Drucker–Prager Cap model	MCC and DCPD * (D = 200 μm)	Investigation of the powder compaction process and prediction of compaction curves for mixtures	The DPC model was predicted using a single material parameter, thus predicting a compaction pressure	It cannot be accurately predicted when particle size varies widely	[27]
Compaction	FEM	Linear elastic model	Tablet parameter (E = 10 GPa, Possion = 0.3)	Investigation of tensile strength of tablets	Modeling selection of convex tablets and capsule-type tablets	Restriction of tablets to linearly elastic and isotropic	[73]
Compaction	FEM	Linear elastic model	ACP, MLac, MCC, MgSt *	Measurement of elastic constants of tablets	The application of impulse excitation techniques to the study of free vibration to assess elastic parameters	Calculation time is long, and the model is complicated	[77]
Decompression	FEM	Elastic model	NaCl, LAC (Size: 19 × 7.6 mm)	Investigation of tensile strength assessment of materials for triaxial decompression	Provides an in-depth understanding of the potential for failure in the tableting	Failure to consider the original strength of the material during decompression	[78]
Compaction	FEM	Drucker–Prager Cap-Perzyna model	MCC (D = 95 μm) LAC (D = 94 μm) AAP * (D = 15 μm)	Investigation of the relationship between compression speed and changes in the internal structure of tablets	Studies were conducted on compaction tests, wireless side compression tests, tensile tests	The compression speeds used in analog are less than those used in industry	[74]
Compaction	FEM	Linear elastic model	Alac, MLac, ACP, MCC, MgSt	Measurement of elastic constants of tablets	The application of pulsed excitation techniques to the study of elastic properties of materials	Restriction of tablets to linearly elastic and isotropic	[79]
Compaction	FEM	Viscoelastic model	Glac, ACP, MCC, Starch, MgSt	Investigation of the relationship between strain rate sensitivity and powder compaction properties	Applying inverse identification of model parameters using the Prony series	Code limitations exist in the simulation	[68]
Compaction	FEM	Drucker–Prager Cap model	MCC (D = 20–200 μm)	Investigation of the causes of tablet failure during compaction was carried out	Studied the compaction process of convex tablets	No analysis of the mechanism of compaction failure	[75]
Compaction	FEM	Drucker–Prager Cap model	MCC, LAC, CS, MgSt (D = 250 μm)	Investigation of the effect of residual stress and tensile strength with disintegration time	The effect of tablet composition on DPC parameters was assessed based on triangular contour diagrams	No elucidation of the role of residual stress at specific sites	[72]
Decompression	FEM	Drucker–Prager Cap model	GLac, ACP, Vc, MgSt	Investigation of the effect of unloading conditions on capping and lamination	Predicted tablet failure in the decompression by capping and lamination	No study of the relationship between residual stress and tablet failure	[12]

* MCC: microcrystalline cellulose, PVP: polyvinyl pyrrolidone, ALac: anhydrous lactose, GLac: granulated lactose monohydrate, SD-Lac: spray-dried lactose monohydrate, ACP: anhydrous calcium phosphate, IsM: isomalt, DCPD: dibasic calcium phosphate dihydrate, MgSt: magnesium stearate, MLac: agglomerated monohydrate lactose, CS: cornstarch, AAP: acetaminophen.

**Table 4:** Combined numerical application of CFD-DEM during compaction.

Process	Numerical Method	Model	Material	Purpose	Advantages	Disadvantages	Reference
Die filling	DEM-CFD *	Continuity equation and Navier–Stokes equations	Coarse grain (D = 125, 250, 375 μm, coarse grain ratio = 1.0, 2.0, 3.0)	Investigation of the filling process in industrial powder die filling	Gas–solid boundary modeling was developed, and extensive particle simulations were performed	No study of the contact behavior of coarse particles with air	[37]
Die filling	DEM-CFD	Continuity and momentum equations, Navier–Stokes equation	Particles (D = 200 μm)	Investigation of the effect of airflow on die filling	Introducing the combination of SDF * and IBM * into the DEM-CFD methodology	Lack of vacuum conditions control experiments	[93]
Die filling	DEM-CFD	Hertz–Mindlin model and continuity and momentum equations	MCC * (D = 50 μm)	Investigation of the effect of suction filling on powder flowability	Using DEM-CFD coupling to investigate the suction-filling process	No consideration of suction filling in the presence of a vacuum	[115]
Die filling	DEM-CFD	Coulomb’s law and Hertz–Mindlin model	Charged particles (D = 100 μm)	Investigation of die filling and flow behavior of electrostatic charges in vacuum and air	First studies of the flow behavior of charged particles under vacuum	Few electrically charged particles in the actual production process	[113]
Die filling	DEM-CFD	Hertz–Mindlin model and continuity and momentum equations	Coarse particles (D_c_ = 360 μm), fine particles (D_f_ = 360 μm)	Investigation of filling and segregation behavior of binary mixtures with different particle sizes in air and vacuum	Realistic 3D modeling was used to study the flow and segregation behavior.	The effect of powder flow and segregation behavior in air is not considered.	[116]
Die filling	DEM-CFD	Continuity and momentum equations	Charged particles (D = 130 μm)	Investigation of filling segregation behavior in the air presence	Simulation of die filling in air and vacuum with CFD and DEM coupling	Lack of influence of other conditions on segregation conditions	[114]

* DEM-CFD: Coupled Discrete Element Method with Computational Fluid Dynamics, SDF: Signed Distance Function, IBM: Immersed Boundary Method, MCC: microcrystalline cellulose.

**Table 5:** DEM and FEM combined with other numerical methods during compaction.

Process	Numerical Method	Contact	Material	Purpose	Advantage	Disadvantage	Reference
Compaction	DEM– Johanson	Linear spring dashpot model and linear spring Coulomb model	Mannitol, MCC, MgSt (D_50_ = 94.4 μm)	Investigation of how to accurately predict the density of bands	Using the DEM with the Johanson model for the prediction of strip density	No studies on the effect of other particle sizes on the compaction process	[121]
Compaction	FEM-MD	The Hill–Mandel principle	Au (Size: 2500 × 500 × 200 μm)	Investigation of the compaction process of metallic nano-powder	Investigating the variation in die wall friction coefficient	No assumptions are made about particle contact	[122]
Compaction	DEM-BPM	An elastic–plastic bonded model and Hertz–Mindlin model	Kaolinite, Albite Quartz (D < 500 μm)	Investigation of the mechanical behavior of uniaxial compaction of ceramic tile particles	Developed mathematical models for bonding behavior, water content, porosity	No research on compaction mechanisms	[123]
Compaction	MC-DEM	Elastic–plastic contact model	MCC (D_10_ = 82.9 μm, D_50_ = 224.6 μm, D_90_ = 379.3 μm)	Investigation of the deformation behavior of non-spherical particles during compaction at high loads	Using the multi-contact DEM and the bonded extreme value method to study deformation behavior	No convergence to the solution, and there is an additional need for BMS method calibration	[120]
Compaction	FEM-MD	Fine-scale (atomistic) model	Nano-powder (Size: 2500 × 500 × 200 μm)	Investigation of the compaction process of nano-powder	Proposed extension of the continuum-atom multiscale method to nano-powders	Contact modeling and calculation methods are complicated	[122]
Compaction	FEM-BEM	Drucker–Prager Cap constitutive model	Al 6061 (D = 0–50 μm)	Investigation of the effect of mechanical properties and microstructure during compaction	Discusses the electromagnetic distribution between the drive coil and the drive plate	No research on compaction mechanisms	[124]
Compaction	FEM-SPH	Elastic–plastic cap model	Gravel, sand, and silty clay layer (size: 4.5 × 6 m)	Investigation of the dynamic compaction process	The three-dimensional model was built using the particle–unit coupling algorithm	Failure to quantify the distance between the hammer head and landing	[125]

## Data Availability

No new data were created or analyzed in this study. Data sharing is not applicable to this article.
